# Coincidence of the facial and maxillary dental midlines in the Peruvian population. A cross-sectional study

**DOI:** 10.4317/jced.61930

**Published:** 2024-09-01

**Authors:** Yéssica Rocío Chahuara-Ramírez, Luis Ernesto Arriola-Guillén

**Affiliations:** 1 MSc student, School of Dentistry, Universidad Científica del Sur, Lima, Perú; 2 Ph.D. and Associate Professor of the Division of Orthodontics, School of Dentistry, Universidad Científica del Sur, Lima, Perú

## Abstract

**Background:**

This study aimed to determine the percentage of perfect and acceptable coincidence between the facial and maxillary dental midlines in individuals from Peru.

**Material and Methods:**

This was a cross-sectional study of a group of Peruvian individuals gathered from private offices in Lima, Peru, from January to June 2024. A total of 279 patients (133 males and 146 females) aged between 18 and 30 were included. We selected patients with permanent teeth up to their second molars, while those with gaps between their front teeth, ongoing or previous orthodontic treatment, or any craniofacial anomalies were excluded. Using a professional camera, we took frontal photographs of the participants at rest and while smiling. The facial morphological index was used to classify facial biotypes into three categories: mesofacial, dolichofacial, and brachyfacial. We then used the PowerPoint program to analyze perfect and accepted coincidence (deviation to either side of up to 2 mm) between the facial and dental midline. The data was analyzed by chi-square tests and binary logistic regression (p>0.05).

**Results:**

Perfect coincidence of the dental midline was present in 23.3% of the cases, while an accepted coincidence was observed in 95.7% of the individuals. When considering facial structure, the percentage of alignment of the maxillary dental midline (perfect or accepted) with the facial midline did not show a significant association (*p*=0.145, *p*=0.870, respectively). Furthermore, there were no significant differences in the percentage of alignment between men and women (*p*=0.241 for perfect coincidence, *p*=0.322 for accepted alignment).

**Conclusions:**

Most Peruvian individuals assessed had an accepted coincidence (up to 2 mm) between the facial midline and the maxillary dental midline, although it is not always perfect. In these cases, orthodontic treatment is needed for optimal occlusal relationships and stable facial esthetics.

** Key words:**Esthetics, Facial midline, maxillary dental midline, Peruvian.

## Introduction

Symmetry has long been essential for harmony and balance in dental and facial esthetics (1,2). The appearance of the face, especially the smile, significantly impacts personal and professional image (3). Orthodontic treatment aims to achieve optimal occlusal relationship and stable facial esthetics (4-7). The position of the facial and dental midlines is crucial for achieving tooth and facial balance. However, slight deviations are accepTable if not noticeable up close, while significant deviations can affect smile attractiveness and esthetics (8,9).

The facial midline is a vertical line that divides a horizontal line drawn from the outer corner of one eye to the outer corner of the other eye. Similarly, the maxillary dental midline is a vertical line drawn through the tip of the incisal embrasure between the two central incisors, parallel to the esthetic frame of the face (10). An accepTable deviation for the maxillary dental midline from the facial midline has been defined as 1.83 mm to 2.91 mm (11).

There are two methods to evaluate the location of the maxillary dental midline. The first requires exact alignment with the facial midline (12-14). In contrast, the second method allows for relative tolerance and considers anatomical structures such as the incisor papilla and labial filtrum (15-17). This second point considers that the midline of the upper teeth can be positioned close to the center of the mouth, within a previously described range of normality (5,18,19). While not necessary, it is preferred for both midlines to align to achieve a balanced dental and lip appearance.

Few studies have evaluated the alignment of the facial midline with the dental maxillary midline, and orthodontists need to know the coincidence percentages for adequate treatment planning (20-24). Therefore, this study aimed to determine the percentage of perfect and accepTable coincidence of the facial and maxillary dental midlines in Peruvian individuals to aid orthodontics to achieve optimal occlusal relationships and facial esthetics.

## Material and Methods

The Institutional Ethics and Research Committee of Cientifica del Sur University approved the following cross-sectional study, with approval code POS-53-2023-00404.

All participants provided informed consent after receiving a detailed explanation of the study. This investigation occurred in three private dental offices in Lima (Peru) from January to June 2024. The study included subjects of both sexes between 18 and 30 years old, with all anterior teeth present and without cavities. Subjects with a history of previous orthodontic treatment, prosthetic treatment of anterior teeth, anterior and posterior teeth extractions, trauma, facial deformities, esthetic jaw surgery or rhinoplasty, severe crowding, and diastemas were excluded. The sample size was determined using a formula to estimate the proportion with data from the previous pilot test, 95% confidence level, 5% precision, and an expected proportion of 90%, resulting in a sample size of 139. However, this study included 279 individuals. https://www.openepi.com/SampleSize/SSPropor.htm

-Procedures

**Figure 1:**
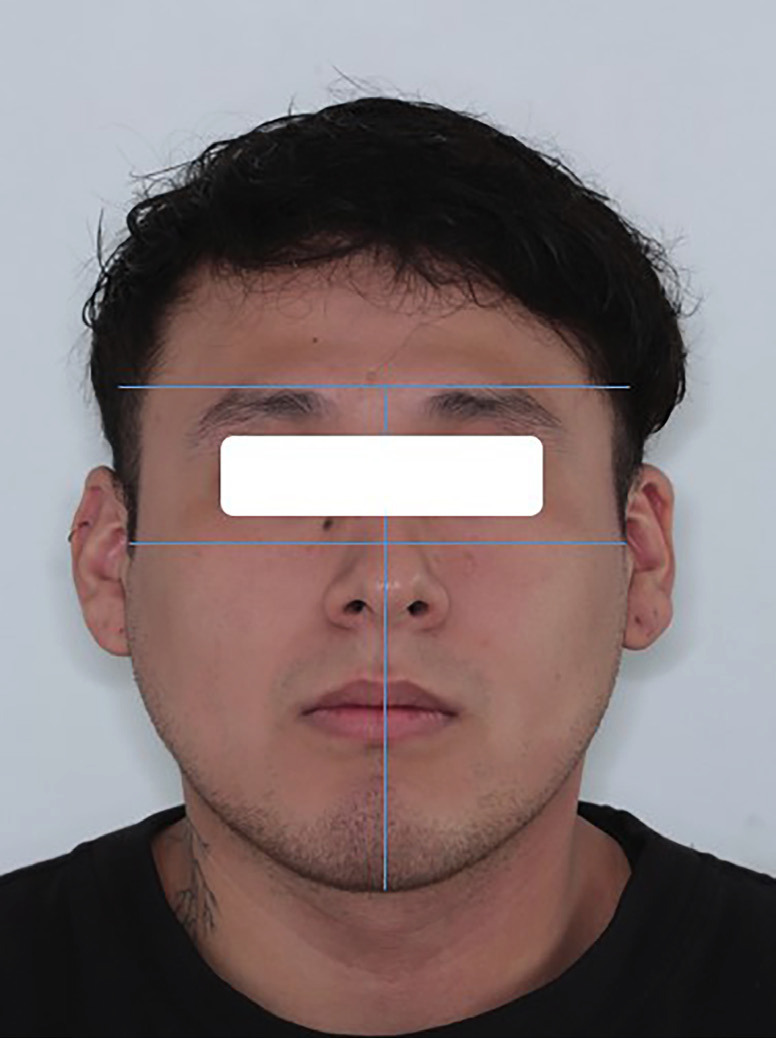
Assessment of facial shape based on the morphological facial index (25).

Frontal photographs were taken with a Canon Rebel T5i and a 100 mm lens, including one at rest and another with a smiling face. Nasal width and lip length were measured using an Ubermann digital vernier caliper. These lengths were also used as a reference to change the size of the images and perform the analysis in the PowerPoint 2013 program. Then, the facial biotype in the resting photograph was assessed and classified into three categories according to the facial morphological index: mesofacial (97°-104°), dolichofacial (> 104°), and brachyfacial (< 97°) (25) (Fig. 1).

**Figure 2:**
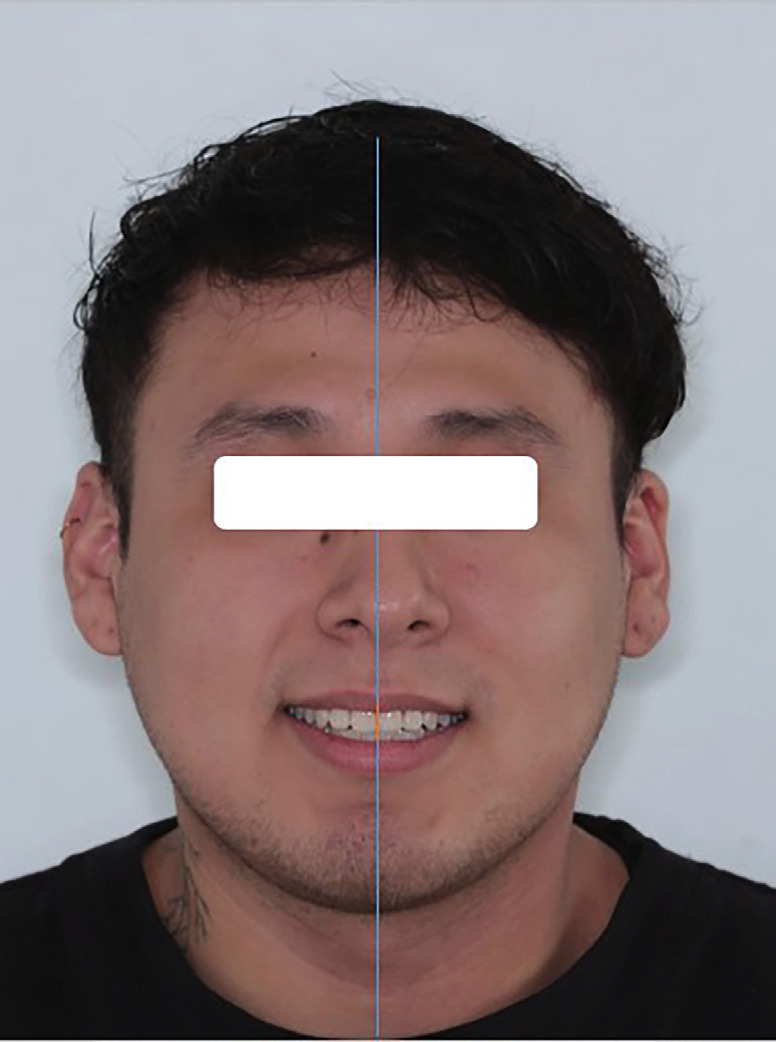
Evaluation of the perfect or accepted coincidence (deviation to either side of up to 2 mm) between the dental and facial midline.

The facial midline was marked in the smiling photograph by drawing a vertical line bisecting a horizontal line drawn across the exocanthion from one eye to the other, following the definition in the glossary of prosthetic terms. This line was related to the maxillary dental midline as the reference to a vertical line drawn across the tip of the incisal embrasure between the two maxillary central incisors, parallel to the vertical lines of the esthetic frame of the face, which was taken as the contact area between the upper central incisors. The PowerPoint program version 11.0 was used to analyze the perfect or accepted coincidence (up to 2 mm of deviation to either side) between the dental midline and the facial midline (11,26,27), (Fig. 2).

-Statistical analysis

The statistical analysis was performed using SPSS version 19.0 for Windows (IBM SPSS, Chicago, Illinois, USA). Initially, the overall percentages of coincidence of both midlines were calculated. Then, the chi-square association test was used to consider sex and facial biotype. Subsequently, binary logistic regression was employed to assess the impact of the predictor variables sex, facial biotype, and age on the occurrence of the exact and accepted coincidence of the upper dental midline. The significance level was set at *P*<0.05.

## Results

 Table 1 shows the baseline characteristics of the sample.  Table 2 shows that exact coincidence of the facial midline with the maxillary dental midline was present in 23.3% of cases. In comparison, accepted coincidence was observed in 95.7% of those evaluated. The percentage of coincidence of the maxillary dental midline (exact and accepted) with the facial midline was also evaluated, considering the facial biotype, with no differences being observed among the three groups compared (*p*=0.145, *p*=0.870, respectively). Similarly, the percentage of coincidences of the maxillary dental midline (exact and accepted) with the facial midline according to sex showed no difference between men and women (*p*=0.241 for exact coincidence, *p*=0.322 for accepted coincidence), ( Table 3, Table 4).  Table 5 shows that in the sample evaluated, the maxillary dental midline mainly deviated to the right side (54.9%). Additionally, when evaluating whether any predictor variable (sex, facial biotype, and age) influenced the alignment of the maxillary dental midline with the facial midline, no variable showed significant influence (*p* >0.05) ( Table 6).

## Discussion

The coincidence of the upper dental midline with the facial midline is considered an essential factor in the esthetics and harmony of a person’s smile (28-32). Consequently, this study aimed to determine the percentage of coincidence between the facial and maxillary dental midlines in Peruvian individuals. It is important to note that although the study was conducted on Peruvians, our results can be applied to adult individuals of different races as there is no biological reason for the deviation of the upper dental midline to have an ethnic origin. Therefore, these results could be valuable for orthodontic practice in general.

In orthodontic practice, it is crucial to diagnose the position of the upper dental midline accurately. Many orthodontists use this parameter as a starting point for treatment planning related to dental intercuspation. There are two recognized methods for evaluating the upper dental midline. The first requires perfect alignment with the sagittal midline of the face (12-14). The second method allows for a deviation of up to 2 mm to either side of the face, referred to in this study as “accepted coincidence” (5,11,18,19,26,27). Orthodontists often use this second diagnostic method because patients view significant deviations as disharmonious and consider dental midline discrepancies to diminish smile attractiveness (8,9). Indeed, it has been reported that there is a 56% probability of discrepancies of 2 mm or more being noticed by laypeople (28), while a lesser midline discrepancy is considered acceptable (30). In this regard, our study found that 23.3% of cases had a perfect coincidence, while 95.7% showed an accepted coincidence. These results are consistent with the findings of Eskelsen *et al*. (30), who found that the interpupillary midline coincided with the upper dental midline in only 38% of the study subjects. Similarly, Maharjan *et al*. (23) reported that most Nepalese individuals had mismatched facial and dental midlines, with only 9.33% showing a perfect coincidence. Miller *et al*. (31) suggested using the labial philtrum as an alternative method to the facial midline. They found that the upper dental midline coincided with the philtrum midline in 75% of cases. Additionally, Melo *et al*. (33) observed that both lines matched in 94.3% of the subjects studied, while Sharma *et al*. (34) found a 72.5% match in the population of Karnataka, India. Lastly, Nold *et al*. (35) examined Caucasian individuals and reported an 85% match between facial and dental midlines.

One of the specific objectives of this study was to determine possible correlations between the coincidence of both midlines based on the facial biotype. The findings showed that there was a perfect coincidence for the mesofacial biotype of 25.6%, being 16.3% for the brachyfacial biotype, and 21.9% for the dolichofacial biotype. Regarding the accepted coincidence, the percentages were 95.3% for mesofacial, 95.3% for brachyfacial, and 96.9% for dolichofacial. No significant association between these values and clinical relevance was found, indicating that the dental midline deviation is indistinct from the facial biotype.

Regarding the exact coincidence based on sex, the study found a 25.3% coincidence in women and a 21.1% coincidence in men, with no significant association found. These results align with the findings of Niraula *et al*. (22), who also observed a slightly more remarkable coincidence of the facial and dental midlines in women than men, but without reaching statistical significance. Although some studies reported that deviation to one side was more frequent than the other, this difference was not deemed clinically relevant. Finally, we found that most individuals in our sample presented an acceptable coincidence within a range of up to 2 mm between the facial midline and the maxillary dental midline. This alignment is generally accepTable, but it may only be precise sometimes.

## Conclusions

In a sample of Peruvian individuals, it was found that alignment between the facial midline and the maxillary dental midline was within an accepted range of up to 2 mm in the majority of subjects, being perfect in less than 25% of the cases. As a result, orthodontists must focus on addressing misalignments to achieve optimal dental and facial symmetry.

## Figures and Tables

**Table 1:** Number of males and females included in the study sample (n=279).

Sex	n	Mean	SD
Male	133	21.62	3.35
Female	146	21.36	3.33

**Table 2:** Percentage of exact and accepted coincidence of the maxillary dental midline with the facial midline.

Type of coincidence	n	%
Perfect coincidence with the facial midline		
No	214	76.7
Yes	65	23.3
Total	279	100.0
Accepted coincidence with the facial midline		
No	12	4.3
Yes	267	95.7
Total	279	100.0

**Table 3:** Association between the exact and accepted coincidence of the maxillary dental midline with the facial midline and facial biotype.

Facial biotype		Perfect coincidence		
		No	Yes	Total	p
Mesofacial	n	128	44	172	0.145
	%	74.4	25.6	100.0	
Brachyfacial	n	36	7	43	
	%	83.7	16.3	100.0	
Dolichofacial	n	50	14	64	
	%	78.1	21.9	100.0	
Total	n	214	65	279	
	%	76.7	23.3	100.0	
Facial biotype		Accepted coincidence		
		No	Yes	Total	p
Mesofacial	n	8	164	172	0.870
	%	4.7	95.3	100.0	
Brachyfacial	n	2	41	43	
	%	4.7	95.3	100.0	
Dolichofacial	n	2	62	64	
	%	3.1	96.9	100.0	
Total	n	12	267	279	
	%	4.3	95.7	100.0	

Chi-square test

**Table 4:** Association between the exact and accepted coincidence of the maxillary dental midline with the facial midline and sex.

Sex		Perfect coincidence		
		No	Yes	Total	p
Male	n	105	28	133	0.241
	%	78.9	21.1	100.0	
Female	n	109	37	146	
	%	74.7	25.3	100.0	
Total	n	214	65	279	
	%	76.7	23.3	100.0	
Sex		Accepted coincidence		
		Not	Yes	Total	p
Male	n	7	126	133	0.322
	%	5.3	94.7	100.0	
Female	n	5	141	146	
	%	3.4	96.6	100.0	
Total	n	12	267	279	
	%	4.3	95.7	100.0	

Fisher exact test

**Table 5:** Side of deviation of the upper dental midline concerning the facial midline.

Side of deviation	n	%
Left	97	45.1
Right	118	54.9
Total	215	100.0

**Table 6:** Binary logistic regression to determine the influence of predictor variables on the exact and accepted coincidence of the maxillary dental midline.

Variables	p	(B)	95% CI	
			Lower	Upper
Perfect coincidence				
Male sex	__	__	__	__
Female sex	0.460	1.238	0.703	2.180
Mesofacial	0.457	___	___	__
Brachyfacial	0.218	0.574	0.238	1.387
Dolichofacial	0.649	0.852	0.427	1.700
Age	0.326	0.956	0.875	1.045
Constant	0.807	0.784	__	__
Accepted coincidence				
Male sex	__	__	__	__
Female sex	0.432	1.605	0.492	5.234
Mesofacial	0.820	___	___	__
Brachyfacial	0.949	1.054	0.214	5.199
Dolichofacial	0.531	1.663	0.339	8.148
Age	0.328	0.925	0.791	1.081
Constant	0.015	87.887	__	__

CI: confidence interval

## Data Availability

The datasets used and/or analyzed during the current study are available from the corresponding author.
